# A Synergistic Dual-Channel Sensor for Ultrasensitive Detection of *Pseudomonas aeruginosa* by DNA Nanostructure and G-Quadruplex

**DOI:** 10.3390/bios13010024

**Published:** 2022-12-26

**Authors:** Wei Yuan, Xinxia Wang, Zhilan Sun, Fang Liu, Daoying Wang

**Affiliations:** 1 Jiangsu Key Laboratory for Food Quality and Safety-State Key Laboratory Cultivation Base, Ministry of Science and Technology, Nanjing 210014, China; 2 Institute of Agricultural Products Processing, Jiangsu Academy of Agricultural Sciences, Nanjing 210014, China; 3 Key Laboratory of Cold Chain Logistics Technology for Agro-Product, Ministry of Agriculture and Rural Affairs, Nanjing 210014, China

**Keywords:** *Pseudomonas aeruginosa*, DNA nanostructure, G-quadruplex, RCA, dual-channel sensor

## Abstract

*Pseudomonas aeruginosa* is one of the foodborne pathogenic bacteria that greatly threatens human health. An ultrasensitive technology for *P. aeruginosa* detection is urgently demanded. Herein, based on the mechanism of aptamer-specific recognition, an electrochemical-colorimetric dual-mode ultrasensitive sensing strategy for *P. aeruginosa* is proposed. The vertices of DNA tetrahedral nanoprobes (DTNPs), that immobilized on the gold electrode were modified with *P. aeruginosa* aptamers. Furthermore, the G-quadruplex, which was conjugated with a *P. aeruginosa* aptamer, was synthesized via rolling circle amplification (RCA). Once *P. aeruginosa* is captured, a hemin/G-quadruplex, which possesses peroxidase-mimicking activity, will separate from the *P. aeruginosa* aptamer. Then, the exfoliated hemin/G-quadruplexes are collected for oxidation of the 3,3′,5′,5′-tetramethylbenzidine for colorimetric sensing. In the electrochemical mode, the hemin/G-quadruplex that is still bound to the aptamer catalyzes polyaniline (PANI) deposition and leads to a measurable electrochemical signal. The colorimetric and electrochemical channels demonstrated a good forward and reverse linear response for *P. aeruginosa* within the range of 1–10^8^ CFU mL^−1^, respectively. Overall, compared with a traditional single-mode sensor for *P. aeruginosa*, the proposed dual-mode sensor featuring self-calibration not only avoids false positive results but also improves accuracy and sensitivity. Furthermore, the consistency of the electrochemical/colorimetric assay was verified in practical meat samples and showed great potential for applications in bioanalysis.

## 1. Introduction

*Pseudomonas aeruginosa* is an opportunistic Gram-negative pathogenic bacterium that plays an important role in food spoilage through proteolytic and lipolytic activities [1,2,3,4,5]. However, traditional detection methods, such as Gram staining and the use of a culture medium, are time-consuming and have low sensitivity; thus, they are not conducive to rapid detection or screening [6]. Therefore, a rapid and sensitive method for detecting this bacterium is urgently needed to reduce its potential harm to humans [7,8].

The vigorous development of DNA nanotechnology has not only invited widespread interest among researchers but has also led to the successful development of related methods for *P. aeruginosa* detection, such as DNA biosensors [7,9], polymerase chain reaction (PCR) [10,11], and loop-mediated isothermal amplification (LAMP) [12,13]. PCR not only requires expensive instruments but also necessitates the training of operators in the use of such instruments. LAMP is highly susceptible to contamination, leading to false-positive results. Therefore, the focus has shifted to the use of biosensors for *P. aeruginosa* detection. Optics sensors [14], colorimetry sensors [7,15,16], and electrochemistry sensors [17,18,19] have been successfully developed for this purpose. A dual-channel sensor for both the colorimetric and electrochemical channels has been developed for the detection of aflatoxin B1 (AFB1) [20]. It will not only substantially improve detection accuracy and diversity but also reduce false-negative and false-positive rates in rapid testing. In the present study, a new mode sensor will be proposed to address the instability of the electrochemistry sensors. This sensor connects the colorimetric and electrochemical sensors.

Based on the exciting potential of DNA technology, various DNA sensors have been successfully constructed for the rapid detection of food contaminants [21,22,23]. Xiong et al. [24] proposed a new strategy based on a DNA tetrahedron nanostructure to improve the sensitivity of AFB1 detection. The limit of detection of this strategy was as low as 0.033 ng mL^−1^. Yuan et al. [25] developed a synergistic strategy based on a DNA tetrahedron nanostructure and hybridization chain reaction (HCR) for ochratoxin A with a detection limit of 0.017 ng mL^−1^. From these results, it was found that the methods based on DNA tetrahedral nanostructures all achieved a high sensitivity. The reasons for these excellent results are as follows: firstly, compared to conventional single-stranded DNA, DNA tetrahedral nanostructures are of a rigid structure that is more stable, which in turn leads to more efficient recognition with the target. Secondly, there are electron mediators in electrochemical sensors that can be attached to the nucleic acid backbone by electrostatic adsorption [26], in situ deposition, etc. The DNA tetrahedral nanostructure itself already presents a high number of nucleic acid backbones, so it can be used as a primary signal amplifier while achieving stability. Crucially, while DNA tetrahedral nanostructures may appear to be more complex than conventional single-stranded DNA, the steps involved are essentially the same (dilution, annealing, and finally, dropping onto the electrode surface to construct the recognition probe). Inspired by this work, a DNA tetrahedron nanostructure is considered in this study. This study aimed to fabricate a dual-channel biosensor via DNA technology. The most critical aspect of this strategy was the selection and design of probes that connect dual channels. Previous works demonstrated that a G-quadruplex can be used as a key component in solving the signaling problem of two channels [27,28,29]. Based on a G-quadruplex/rolling circle amplification (RCA) strategy, Tang et al. [30] proposed an electrochemical sensor for microRNA detection. Through RCA, numerous free G-rich sequences were synthesized. Subsequently, with the presence of K^+^, G-quadruplexes were folded by the free G-rich sequences that could capture methylene blue (MB) to substantially improve the electrical signal. Ultimately, its detection limit was 2.75 fM. In the presence of hemin, it was inserted into a G-quadruplex to form a hemin/G-quadruplex, which displayed peroxidase-mimicking activity. In the presence of hydrogen peroxide (H_2_O_2_), it can not only catalyze the color change of 3,3′,5,5′-tetramethylbenzidine (TMB) and thiamine but also promote the deposition of aniline [31,32,33].

A DNA probe was proposed in this study, which can not only reduce the hindrance and improve accessibility but also provide a basis for the novel dual-channel sensor for the efficient detection of *P. aeruginosa*. Owing to the superiority of the DNA probes, more hemin/G-quadruplexes were conjugated via RCA in the electrochemical mode. It is an excellent peroxidase mimic that can catalyze the formation of PANI with electrical signals from aniline in a mild environment. A template is required for the deposition of aniline, whereas the DNA probes and G-quadruplexes conjugated via RCA can provide an abundant number of negatively charged phosphate backbones as a template. Owing to the negative correlation between *P. aeruginosa* and the hemin/G-quadruplexes, high-sensitivity detection was accomplished. The hemin/G-quadruplexes that failed still exhibited catalytic activity, and they were able to oxidize TMB to produce color changes in the presence of H_2_O_2_. Therefore, the hemin/G-quadruplexes that were not involved in the reaction were collected, and their use facilitated naked-eye detection in colorimetric mode. The results of the dual-channel sensor can corroborate each other and improve the stability of the sensor. Moreover, the colorimetric sensor can be used to detect *P. aeruginosa* via the naked eye to improve the sensor’s detection efficiency. A novel dual-channel sensor based on electrochemical and colorimetric sensors was successfully designed.

## 2. Materials and Methods

### 2.1. Materials and Apparatus

The oligonucleotides in this work (Table 1), DNA marker, PBS, 5 × TAE buffer, dNTPs, T4 DNA ligase, exonuclease I (Exo I), and exonuclease III (Exo III) were purchased from Shanghai Sangon Biological Engineering Technology & Services Co. Ltd. (Shanghai, China). The oligonucleotides are listed in Table S1. *P. aeruginosa*, *P. putida*, *P. fluorescens*, *Escherichia coli*, and *Clostridium perfringens* were preserved in our laboratory.

The hemin, aniline, hydrogen peroxide (H_2_O_2_), dimethyl sulfoxide, tris(2-carboxyethyl) phosphine hydrochloride (TCEP), TMB, and 6-mercapto-1-hexanol were bought from Aladdin Industrial (Shanghai, China). Phi29 DNA polymerase was obtained from New England Biolabs (Ipswich, MA, USA). The samples (chicken, duck, pork, beef, and mutton) were acquired from a local market in Nanjing, China.

The buffer solutions utilized in this study were as follows: 1 × PBS; 1 × T4 ligase reaction buffer (40 mM Tris-HCl, pH 7.8, 10 mM MgCl_2_, 10 mM DTT, 0.5 mM ATP); 1 × phi29 polymerase buffer (50 mM Tris-HCl, pH 7.5, 10 mM (NH_4_)_2_SO_4_, 10 mM MgCl_2_, 4 mM DTT); TE buffer (10 mM Tris, pH 8.0, 1 mM EDTA); TM buffer (20 mM Tris, pH 8.0, 50 mM MgCl_2_); TK buffer (20 mM Tris-HCl, pH 8.0, 50 mM KCl, 2 μM hemin); deposition buffer (100 mM acetic acid–sodium acetate (HAc-NaAc), pH 4.3, 200 mM aniline, 20 mM H_2_O_2_, 50 mM KCl, prepared daily); electrolyte (100 mM HAc-NaAc buffer, pH 4.3); electrochemical impedance spectroscopy (EIS) buffer (1 × PBS, 5 mM K_3_[Fe(CN)_6_]/K_4_[Fe(CN)_6_] (1:1), 100 mM KCl); and chromogenic buffer (20 mM TMB, 20 mM H_2_O_2_, 1 × PBS). Ultrapure water (18.2 MΩ cm^−1^ at 25 °C) from a pure water system (GWA-UN1-F40, Persee, China) was used in all experiments.

A model CHI760E electrochemistry workstation (Shanghai Chenhua Instruments Co., Ltd., Shanghai, China) was employed for all electrochemical experiments. The morphology of PANI was analyzed via scanning electron microscopy (SEM) (EVO-LS10 SEM, Carl Zeiss AG, Jena, Germany). UV–Vis spectra were recorded using an Epoch Microplate Spectrophotometer (BioTek Instrumentals, Winooski, VT, USA). 

A characteristic absorption peak of oxidized TMB at 652 nm was used for the quantitative analysis. All measurements were implemented at room temperature.

### 2.2. Fabrication of the DNA Probe on Gold Electrode

Following previous protocols [34,35], the DNA probe was self-assembled and modified on a gold electrode (2 mm in diameter) with Au-S. First, four strands (Tetra-1, Tetra-2, Tetra-3, and Tetra-4) were dissolved in TE buffer, yielding a final concentration of 50 μM. Then, 1 µL of each strand and TCEP (500 mM) were combined with 45 μL of TM buffer. The mixture was heated at 95 °C for 10 min and then cooled at 4 °C for 30 min to form the DNA tetrahedron structure. On top of the structure was a part of the sequence that could be complementary to the target sample. Afterwards, 20 μL of the DNA tetrahedron structure was added to the gold electrode, cleaned [36], and left to react overnight at room temperature. Thus, the DNA probe was successfully fabricated on the gold electrode.

### 2.3. Implementation of RCA

#### 2.3.1. Preparation of Circular DNA Template

A circular DNA template was synthesized using an RCA template, a primer, and a T4 DNA ligase. Afterward, 10 μL of the RCA template (100 μM) and 5 μL of the primer (100 μM) were mixed in 1 × T4 ligase reaction buffer. The solution was heated to 95 °C for 5 min and then slowly cooled to room temperature to induce hybridization. Subsequently, 10 μL of the T4 DNA ligase was added. The mixture was incubated at 16 °C overnight and stopped by heating at 65 °C for 10 min. Uncirculated single-stranded DNA or nonspecifically hybridized dsDNA was eliminated by separately adding 5 U of Exo I and 5 U of Exo III. The system was held at 37 °C for 6 h and stopped by heating at 80 °C for 20 min. Eventually, a circular DNA template was prepared.

#### 2.3.2. Synthesis of G-Quadruplex

The synthesis of the G-quadruplex depended on RCA. A traditional RCA reaction was performed in 100 μL of a solution containing 10 μL of the circular DNA template, capture probe (5 μM), phi29 DNA polymerase (10 U), dNTPs (2.5 mM), and BSA (100 μg mL^−1^) in 1 × phi29 polymerase buffer at 30 °C for 3 h. The synthesis generated large G-rich oligonucleotide sequences, which formed the G-quadruplex.

### 2.4. Establishment of Dual-Channel Detection Platform

Before the RCA reaction solution was conjugated in the DNA probe at room temperature overnight, the modified electrodes were cleaned three times with 1 × PBS. The hemin/G-quadruplex DNAzyme was formed by rinsing the electrode with TK buffer and then immersing it in TK buffer at room temperature for 2 h. Subsequently, different concentrations of *P. aeruginosa* were added to the electrode at room temperature for 2 h to compete with the G-quadruplex sequence for binding.

#### 2.4.1. Electrochemical Biosensor Platform

After the electrode was rinsed with electrolyte buffer three times, the gold electrode was immersed in the deposition buffer at room temperature for 2 h. Eventually, a saturated calomel electrode and a platinum wire electrode were used as the reference electrode and the auxiliary electrode, respectively. Their electrochemical signal was measured by performing cyclic voltammetry (CV) and differential pulse voltammetry (DPV) by scanning them from −0.2 to 0.2 V in an electrolyte buffer. Electrochemical impedance spectra (EIS) were recorded in EIS buffer within the frequency range of 0.01–10^5^ Hz.

#### 2.4.2. Colorimetric Biosensor Platform

The reaction solution used for the electrode was retained in a centrifuge tube. Subsequently, a chromogenic buffer was mixed. After 8 min at room temperature, the reaction was measured according to the UV–Vis spectra. Eventually, the color change results could not only be detected but were also observable with the naked eye.

The two sensors (single DNA + RCA and DNA probe + G4) were compared. The sensors were fabricated following the same steps.

### 2.5. Agarose Gel Electrophoresis

The implementation of the DNA probe and the RCA/G-quadruplex was verified by performing all the DNA processing methods as per the above steps for later use. Then, the probe and the quadruplex were analyzed with 2% agarose in 1 × TAE buffer at a constant voltage of 110 V at room temperature for 40 min and then imaged with a gel imaging system (1600, Tanon, Shanghai, China).

### 2.6. Detection of Real Samples

The authenticity of our design was proved by comparing two different detection methods, namely the traditional bacterial counting method and the method described in this paper. Meanwhile, different pretreatments were used. Chicken, duck, pork, beef, and mutton samples were used as mode samples. About 1 mL of 10^3^ CFU/mL *P. aeruginosa* suspension was inoculated into each 10 g of meat sample according to our previous paper [37]. For the traditional bacterial counting method, *P. aeruginosa* was counted using a selective *Pseudomonas* CFC medium.

## 3. Results and Discussion

### 3.1. Principle of Dual-Channel Biosensor

**Scheme 1:**
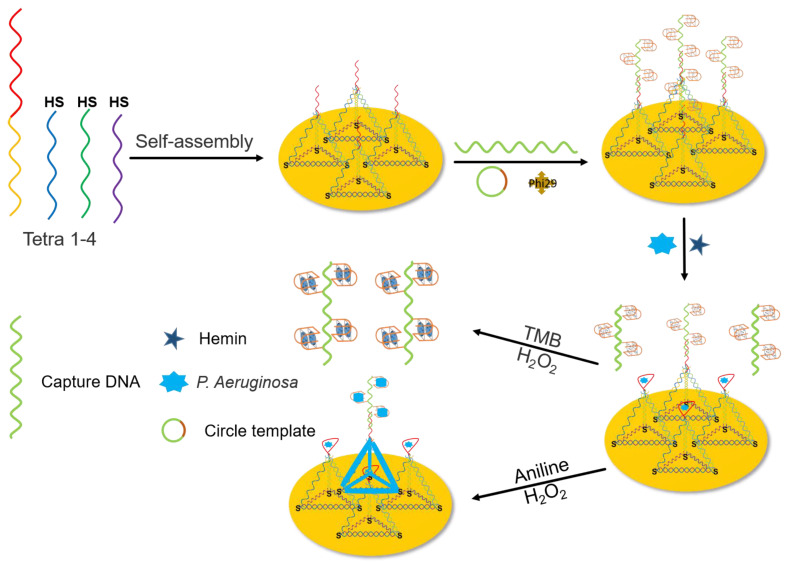
A synergistic dual-channel sensor for *P. aeruginosa* detection.

In this work, a synergistic dual-channel biosensor (Scheme 1) based on a DNA nanostructure and a G-quadruplex was prepared. A stable and ordered DNA tetrahedron structure with aptamers on the top was constructed on a gold electrode with Au-S. Interestingly, the aptamer on the top was partially complementary to the RCA. In the presence of *P. aeruginosa*, they competed together to bind the aptamers to the gold electrode to form the DNA probe. Owing to the dual platform, *P. aeruginosa* was accurately and sensitively detected. In the electrochemical sensor channel, the hemin/G-quadruplex was conjugated on the DNA probe. Subsequently, aniline was deposited on the DNA probe in situ via hemin/G-quadruplex catalysis, thereby successfully forming PANI. Ultimately, owing to the electrical activity of PANI, the signal of *P. aeruginosa* was converted into an electrical signal to achieve sensitive detection. Surprisingly, although the hemin/G-quadruplex failed in the competitive reaction, its activity persisted and was not affected. Therefore, a colorimetric sensor channel was proposed. This sensor could mutually verify the signal of *P. aeruginosa* with the electrochemical sensor to improve the detection accuracy and efficiency. In the colorimetric sensor channel, oxidation of TMB by the hemin/G-quadruplexes was considered as the critical step. After the competitive reaction, the solution on the gold electrode was collected. Immediately, H_2_O_2_ and TMB were separately added. After 8 min, color change was observed, and the absorbance was recorded with a microplate reader. A dual-channel sensor with the advantages of simple operation, low-cost, and time-saving was successfully constructed. This sensor is attractive for monitoring.

### 3.2. Characterization of the DNA Probe/G-Quadruplex

**Figure 1:**
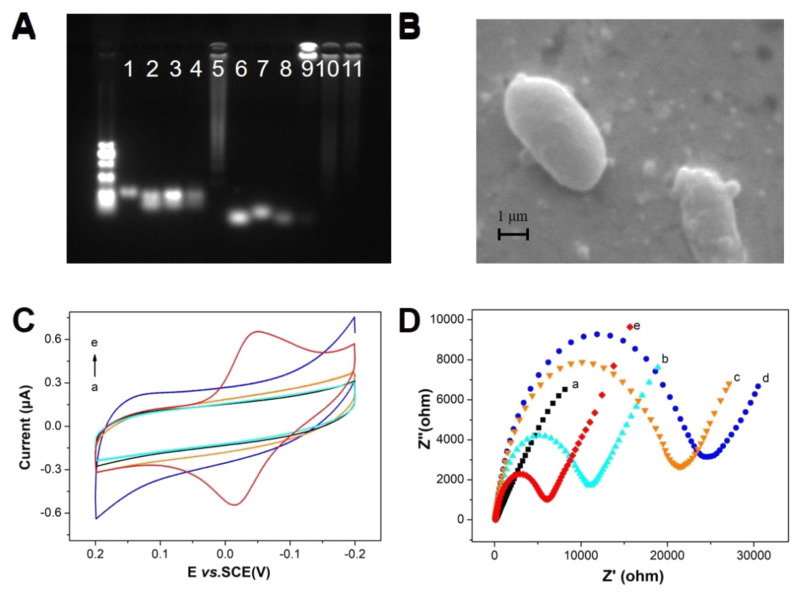
(**A**) Agarose gel electrophoresis image. Lane 1, 10 μM Tetra−A; lane 2, 10 μM Tetra−B; lane 3, 10 μM Tetra−C; lane 4, 10 μM Tetra−D; lane 5, DNA tetrahedron structure; lane 6, 10 μM RCA template; lane 7, 10 μM primer; lane 8, 10 μM capture; lane 9, hemin/G−quadruplexes; lane 10, DNA tetrahedron structure + hemin/G−quadruplexes (lane 5 mixture with lane 9 for 2 h); lane 11, DNA probe + hemin/G-quadruplexes + *P. Aeruginosa* (lane 5, lane 9 and lane 10 mixture for 2 h). (**B**) SEM image of PANI/*P. Aeruginosa* modified gold electrode. (**C**) Cyclic voltammograms and (**D**) Nyquist plots of (a) bare gold electrode, (b) DNA probe, (c) hemin/G−quadruplexes/DNA probe, (d) *P. aeruginosa*/hemin/G−quadruplexes/DNA probe, (e) PANI.

In a typical determination process, agarose gel electrophoresis serves as one of the technical methods for verifying the structure of nucleic acid strands. As shown in 1a, the positions of lanes one, two, three, four and five indicate the successful self-assembly of the DNA probe. Lanes six, seven, eight, and nine indicate the superiority of the RCA. When the DNA probe and RCA were combined by base-pairing rules, a new complex was formed, causing lane 10 to remain unmoved. At the same time, in the presence of *P. aeruginosa*, lane 11 also remained on top without remarkable changes [38,39]. SEM (Figure 1b) was performed to prove that the process was successful. When *P. aeruginosa* was captured by the DNA probe, the rod-shaped structure of *P. aeruginosa* and the particles of PANI were observed [40]. Thus, the sensor was deemed to have been perfectly assembled. CV and EIS are usually treated as techniques for evaluating the assembly process of sensors. A pair of obvious oxidation peaks and reduction peaks at −0.052 and −0.015 V could be observed in Figure 1c. These peaks are one of the characteristics that indicate that PANI has formed. As the assembly progressed, the impedance continued to increase (Figure 1d). However, when the assembly was completed, the impedance was greatly reduced because of the superior conductivity of PANI. Obviously, this trend is also another feature of PANI [24]. Further experiments showed that the sensor is a diffusion control process (Figure S1 in Supplementary Materials).

### 3.3. Comparison of Different Sensing Strategies

**Figure 2:**
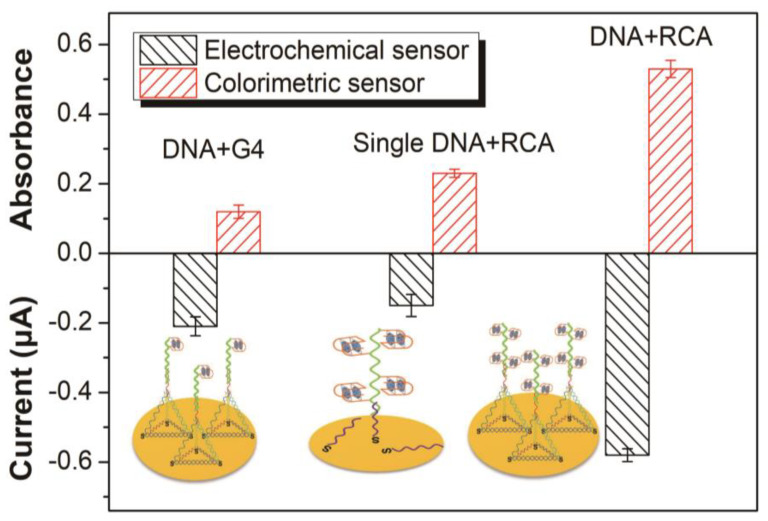
*P. aeruginosa* analysis results from three different sensors: DNA + G4, Single DNA + RCA, and DNA + RCA.

Herein, the uniqueness of the design and the superiority of the sensor detection developed and two sensors (a single DNA + RCA and a DNA probe + G4) were compared (Figure 2). Compared to the single DNA + RCA sensor, the detection performance of the electrochemical sensor and the colorimetric sensor was improved by four- and threefold in the DNA probe + RCA, respectively. Hence, the assembly of the single DNA was disordered and unstable on the gold electrode. Furthermore, the conjugation effect of RCA was difficult to guarantee. Therefore, the effect of the single DNA + RCA sensors was substantially reduced. Compared to the DNA probe + G4, the detection performance of the dual-channel sensor was considerably improved three- and fivefold. Although the DNA probe was stable and assembled in an orderly way on the gold electrode, the lack of RCA meant a large number of hemin/G-quadruplexes were unavailable to delay or even hinder the formation of PANI. Correspondingly, the colorimetric sensor lacks some key components (RCA), which results in a significant reduction in the catalytic effect of the colorimetric process. From the results, an approximately five-fold increase in sensitivity was found after RCA. The dual-channel sensor draws on the advantages of the DNA probes and RCA. The stable and orderly characteristics of the DNA probe were used to construct electrochemical sensors. RCA served as a bridge for communication [41]. The results showed that the sensitivity of this method was greatly improved.

### 3.4. Dual-Channel Detection of P. aeruginosa

**Figure 3:**
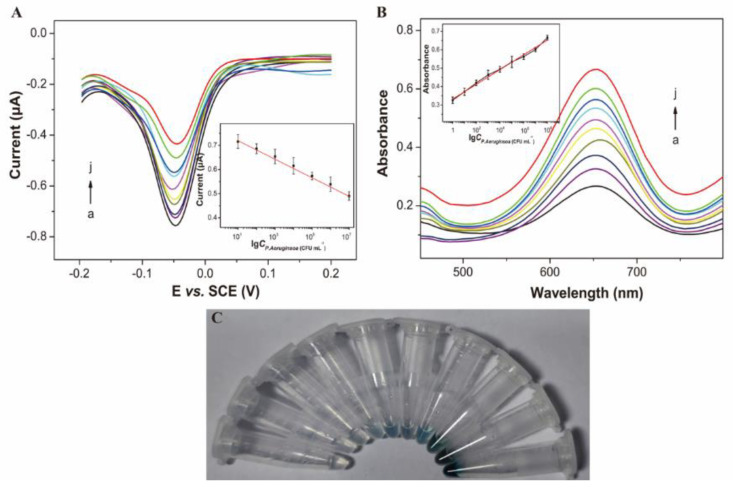
(**A**) Electrochemical sensor for detection of *P. aeruginosa* at different concentrations. (**B**) Colorimetric sensor for detection of *P. aeruginosa* at different concentrations. (**C**) Photograph of the colorimetric sensor in the presence of different *P. aeruginosa* concentrations. From a to j: 0, 1, 10^1^, 10^2^, 10^3^, 10^4^, 10^5^, 10^6^, 10^7^, and 10^8^ CFU mL^−1^. The inset shows the linear relationship between the respective peak intensity and the patina concentration between 10–10^7^ CFU·mL^−1^ and 1–10^8^ CFU·mL^−1^, respectively. The photo was collected after 8 min of reaction.

The ideal calibration curves (Figure 3) of the electrochemical sensor and the colorimetric sensor were plotted under optimal conditions (Figure S2). Owing to the competitive reaction, when the analyte concentration was low, a large amount of RCA was conjugated to the DNA probe. Furthermore, a large amount of aniline was deposited on the backbone of the nucleic acid chain of the probe, which produced PANI and was recognized by DPV. Finally, the ultrasensitive detection of *P. aeruginosa* was achieved through negative correlation. In the electrochemical channel (Figure 3a), the electrochemical biosensor used to sense *P. aeruginosa* quickly has a good relationship in the range of 10–10^7^ CFU mL^−1^ with a limit of detection (LOD) of 1.7 CFU mL^−1^ (inset in Figure 3a). By contrast, the colorimetric detection had a positive correlation because of the superior sensitivity of the color reaction. Eventually, the colorimetric channel (Figure 3b), used to sense *P. aeruginosa* also showed a good linear relationship in the range of 1–10^8^ CFU mL^−1^ with a LOD of 1.3 CFU mL^−1^ (inset in Figure 3b). The results of the stability experiments (Figure S5) demonstrated that a photograph of the reaction could be captured using a mobile phone after 8 min (Figure 3c). The results clarified that the concentration of *P. aeruginosa* was positively correlated with color, thereby facilitating the detection of the bacterium with the naked eye. The excellent detection performance of the dual-channel sensor was verified by comparing its sensitivity to that of other methods (Table 2). The results suggested that the sensor developed herein had high sensitivity.

### 3.5. Selectivity

**Figure 4:**
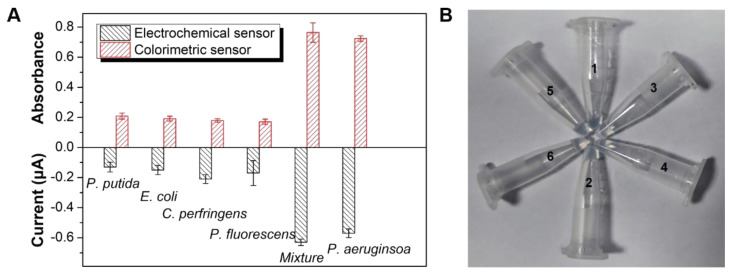
Sensor selectivity. (**A**) The detecting signals of dual-channel sensors. (**B**) Photograph of reaction solution for colorimetric sensing. From 1 to 5: *P. aeruginosa*, mixture, *P. putida*, *E. coli* and *C. perfringens*, *P. fluorescens*. *P. aeruginosa*: 10^2^ CFU mL^−1^, others: 10^8^ CFU mL^−1^, 10^2^ CFU mL^−1^ *P. aeruginosa*, and others 10^8^ CFU mL^−1^ used as mixture. The photo was collected after 8 min of reaction.

Selectivity is the focus of the development of this method. The selectivity of the sensor to *P. putida*, *E. coli*, *C. perfringens*, *P. fluorescens*, and mixed samples was tested and compared with that of *P. aeruginosa*. As shown in Figure 4a, even if the concentration of the other bacteria was 10^8^ CFU mL^−1^, no notable change in the current signal and absorbance value could be observed. The same observation was noted in the mixed sample. As shown in Figure 4b, none of the samples showed a color reaction, except for *P. aeruginosa* and the mixed sample. Therefore, the specificity of the sensor proposed herein met expectations.

### 3.6. Analysis of P. aeruginosa in Meat Samples

The ultimate goal of sensor design is to achieve practical application. The performance of the sensor in detecting *P. aeruginosa* in five meat samples, namely, chicken, duck, pork, beef, and mutton, was compared with that of a traditional method, namely, the plate colony-counting method. Before the experiment, *P. aeruginosa* was counted for additional experiments. As shown in Table 3, the recovery range of *P. aeruginosa* was 81.2–116.4% and 90.7–106.8% using the electrochemical and colorimetric detection channels, respectively. Given that the selective medium is not specific, *P. aeruginosa* cannot be selectively detected via traditional methods. The total number of *P. aeruginosa* detected by a selective medium in other meat samples was much higher than the actual number added, and only the number of *P. aeruginosa* detected in beef was similar to the added. Therefore, the sensitivity of the traditional plate counting method is poor.

## 4. Conclusions

A new biosensing strategy that uses hemin/G-quadruplexes as a bridge to communicate between dual channels was developed and used to detect *P. aeruginosa*. The hemin/G-quadruplexes play an extremely important role in this strategy. They not only deposit electroactive polyaniline in situ in the electrochemical channel but also catalyze the color reaction of TMB in the colorimetric channel. The experimental results of the two channels were compared. The detection accuracy was greatly improved via this mode, and the bacterium could be detected with the naked eye. The detection limits of both sensors were as low as 1 CFU mL^−1^. The sensors performed well in real sample analysis. This strategy can be expanded to other food contaminants. Moreover, dual-mode sensors can be developed on the basis of multiple color luminescence.

## Figures and Tables

**Table 1:** The sequences for this work.

Sequence	5′-3′
Tetra-1	CCCCCGTTGCTTTCGCTTTTCCTTTCGCTTTTGTTCGTTTCGTCCCTGCTTCCTTTCTTG-AAAAA-ACATTCCTAAGTCTGAAACATTACAGCTTGCTACACGAGAAGAGCCGCCATAGTA
Tetra-2	HS-(CH2)6-TATCACCAGGCAGTTGACAGTGTAGCAAGCTGTAATAGATGCGAGGGTCCAATAC
Tetra-3	HS-(CH2)6-TCAACTGCCTGGTGATAAAACGACACTACGTGGGAATCTACTATGGCGGCTCTTC
Tetra-4	HS-(CH2)6-TTCAGACTTAGGAATGTGCTTCCCACGTAGTGTCGTTTGTATTGGACCCTCGCAT
Single DNA	CCCCCGTTGCTTTCGCTTTTCCTTTCGCTTTTGTTCGTTTCGTCCCTGCTTCCTTTCTTG-AAAAA-(CH2)6-SH
RCA template	PO43-TTCAGGTAGTGCATCACCCTCCCACCCCTCCCACCCCTATATCGGAGC
Capture probe	GCGAAAGCAACGGGGCACTACCTGAAGCTCC

**Table 2:** Comparison of sensitivity for *P. aeruginosa* detection.

Method	System	Detection Range	LOD	Reference
Electrochemical	A biosensor based on the aptamer immobilization on the surface of engineered ZIFs-8 via carbodiimide cross-linking	12–1.2 × 10^7^ CFU mL^−1^	1 CFU mL^−1^	[42]
Fluorescence	Graphene Oxide Quantum Dots Assisted Fluorescent Aptasensor	1.28 × 10^3^–2 × 10^7^ CFU mL^−1^	100 CFU mL^−1^	[43]
bioluminescence	firefly luciferase-adenosine triphosphate bioluminescence system	600–3.0 × 10^5^ CFU mL^−1^	200 CFU mL^−1^	[44]
Fluorescence	Novel Pyrimidine Tagged Silver Nanoparticle-Based Fluorescent Immunoassay	8–10^–1^ CFU mL^−1^	1.5 CFU mL^−1^	[1]
Electrochemical	An immunosensor using ZIF-8/Au NPfilm as a platform	10^1^–10^5^ CFU mL^−1^	3.53 CFU mL^−1^	[45]
Electrochemical/colorimetric	Dual channel sensor	10–10^6^/1–10^8^ CFU mL^−1^	1.7/1.3 CFU mL^−1^	This work

**Table 3:** Detection ability of *P. aeruginosa* biosensor in real samples.

Sample	Added (CFU g^−1^)	Traditional Plate Count		The Proposed Dual-Mode Biosensor				
				Electrochemical Sensor		Colorimetric Sensor		
		Found (CFU g^−1^)	Recovery (%)	Found (CFU g^−1^)	Recovery (%)	Found (CFU g^−1^)	Colorimetric Images	Recovery (%)
Chicken	99	>10^2^	-	108.1	109.2	94.7	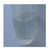	95.6
Duck	112	>10^2^	-	90.9	81.2	101.6	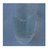	90.7
Pork	103	>10^2^	-	113.5	110.2	97.5	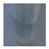	94.7
Beef	86	>10^2^	-	100.1	116.4	89.5	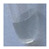	104.4
Mutton	94	109	115.9	87.5	93.1	100.4	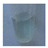	106.8

## Data Availability

The data presented in this study are available in supplementary material.

## Supplementary Materials

The following supporting information can be downloaded at: https://www.mdpi.com/article/10.3390/bios13010024/s1, Figure S1: (A) CVs of the PANI/gold electrode in electrolyte solution at different scan rates of (a) 20, (b) 50, (c) 100, (d) 150, (e) 200, (f) 250, (g) 300, (h) 350, (i) 400, (j) 450, and (k) 500 mV s^−1^. (B) The plots of peak current versus the square foot of the scan rate. 102 CFU mL^−1^ *P. aeruginosa* was selected. Figure S2: (A), (B), and (C) represent the influence of H_2_O_2_ (10, 20, 30, 40, and 50 mM) Time (30, 60, 90, 120, 150, 180, and 210 min), and Aniline (50, 100, 150, 200, and 250 mM) in electrochemical sensor, respectively. (D), (E), and (F) represent the influence of H_2_O_2_ (0, 10, 30, 50, and 70 mM), TMB (0.3, 0.5, 1, 3, and 5 mM), and Hemin (10, 30, 50, 70, 100, and 150 mM) in colorimetric sensor, respectively. 10^2^ CFU mL^−1^ *P. aeruginosa* was used. Error bars showed the standard deviation of three experiments. Figure S3: (A) Colorimetric sensor (B) Electrochemical sensor for detection of *P. aeruginosa* at different concentrations. *P. aeruginosa* analysis results from three different sensors: DNA+G4 (a), Single DNA+RCA (b), and DNA+RCA (c). Figure S4: The insets in Figure 4a,b show the linear relationship between the respective peak intensity and the patina concentration between 1 and 10^8^ CFU·mL^−1^. Figure S5: The relationship between absorbance and time in the colorimetric sensor. References [46,47] are cited in Supplementary Materials.
