# Supplementary material for: A Synergistic Dual-Channel Sensor for Ultrasensitive Detection of Pseudomonas aeruginosa by DNA Nanostructure and G-Quadruplex
Source: Biosensors (Basel). 2022 Dec 26;13(1):24. doi: 10.3390/bios13010024 (PMC9856186; doi:10.3390/bios13010024)
Supplement: Supplementary file 1 [file biosensors-13-00024-s001.zip › biosensors-2051946-supplementary.pdf]

biosensors

MDPI

Article

A Synergistic Dual-Channel Sensor for Ultrasensitive Detection of *Pseudomonas aeruginosa* by DNA Nanostructure and G-quadruplex

Wei Yuan [1,2,†], Xinxia Wang [1,2,†], Zhilan Sun [1,2,3], Fang Liu [1,2,*] and Daoying Wang [3,*]

[1] Jiangsu Key Laboratory for Food Quality and Safety-State Key Laboratory Cultivation Base, Ministry of Science and Technology, Nanjing 210014, China
[2] Institute of Agricultural Products Processing, Jiangsu Academy of Agricultural Sciences, Nanjing, 210014, China
[3] Key Laboratory of Cold Chain Logistics Technology for Agro-product, Ministry of Agriculture and Rural Affairs, Nanjing 210014, China
* Correspondence: fangliu82@163.com (F.L.); daoyingwang@yahoo.com (D.W.)
† These authors contributed equally to this work.

S1. Diffusion of the Biosensor

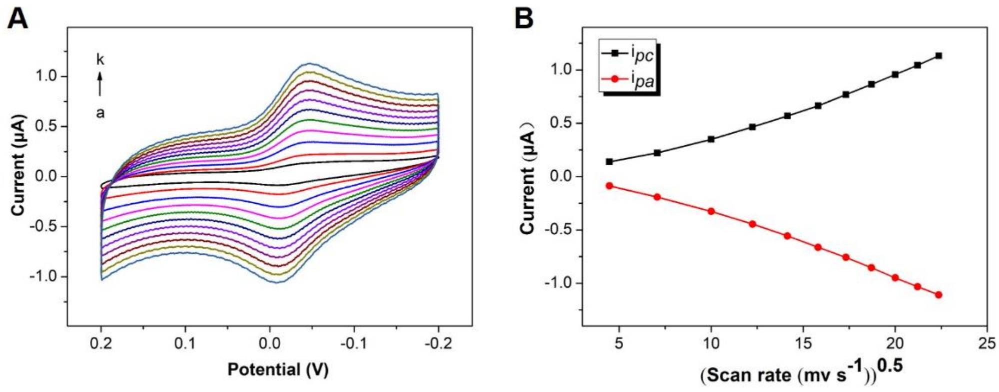

Figure S1. (A) CVs of the PANI/gold electrode in electrolyte solution at different scan rates of (a) 20, (b) 50, (c) 100, (d) 150, (e) 200, (f) 250, (g) 300, (h) 350, (i) 400, (j) 450, and (k) 500 mV s^{-1}. (B) The plots of peak current versus the square foot of the scan rate. 102 CFU mL-1 P. aeruginosa was selected.

To investigate the process of this sensor, the scan rate dependence on the response of PANI/gold electrode was used (Fig. A1). The scan rate was not only positively correlated with the oxidation peak and reduction peak, but also the peak response of the anode and cathode was proportional to its square root. This phenomenon illustrates that the sensor is a diffusion control process [1].

S2. Optimization of the Dual-channel Sensor

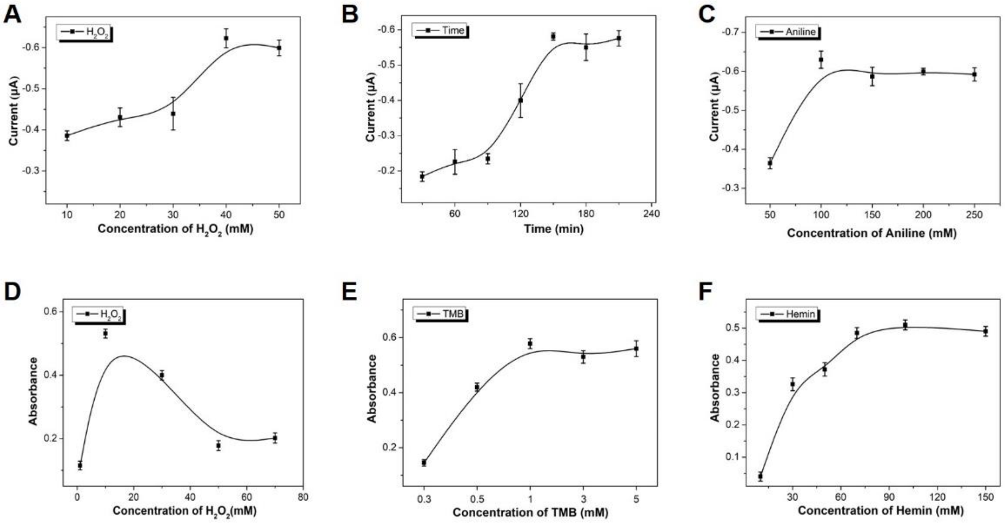

Figure S2. (A), (B), and (C) represent the influence of H_2O_2 (10, 20, 30, 40, and 50 mM) Time (30, 60, 90, 120, 150, 180, and 210 min), and Aniline (50, 100, 150, 200, and 250 mM) in electrochemical sensor, respectively. (D), (E), and (F) represent the influence of H_2O_2 (0, 10, 30, 50, and 70 mM), TMB (0.3, 0.5, 1, 3, and 5 mM), and Hemin (10, 30, 50, 70, 100, and 150 mM) in colorimetric sensor, respectively. 10^2 CFU mL^{-1} *P. aeruginosa* was used. Error bars showed the standard deviation of three experiments.

S2.1 Electrochemical Biosensor

In electrochemical biosensor, the formation of polyaniline (PANI) was a decisive factor. Therefore, some factors that affect aniline deposition were considered, such as H_2O_2, reaction time, and aniline.

On the one hand, H_2O_2 promotes the deposition of aniline, on the other hand, excessive H_2O_2 causes hemin/G-quadruplexes inactivation, thereby reducing the catalytic effect. Therefore, the concentration of H_2O_2 was verified (Fig S2A). How to obtain accurate results in the shortest time was the goal of struggle. When the reaction reaches a certain time, only by extending the time, no substantial progress can be obtained (Fig S2B). Conversely, it increases the cost of testing. Obviously, the concentration of aniline plays an important role. However, the negatively charged phosphate backbone provided by the DNA probe and RCA was definite, too much aniline cannot continue to be deposited (Fig S2C). In view of the above results, 40 mM H_2O_2, 150 min, and 100 mM aniline was selected [2,3].

S2.2 Colorimetric Biosensor

From Fig S2D, when the concentration of H_2O_2 was 10 mM, the effect was best. The reason for result was that excessive H_2O_2 affects the activity of hemin/G-quadruplexes. TMB was key factor in colorimetric sensor. However, when the concentration exceeds 1 mM, there is no significant change from fig S2E. This may be because excessive TMB cannot participate in subsequent reactions. There is no substitute for the catalytic effect of hemin/G-quadruplexes. When hemin was embedded in G-quadruplex, it was really formed. Therefore, to ensure that G-quadruplexes were used, different concentrations of hemin were considered (Fig S2F). In summary, 10 mM H_2O_2, 1 mM TMB, and 70 mM hemin was selected [4,5].

S3. Comparation of Different Sensing Strategies

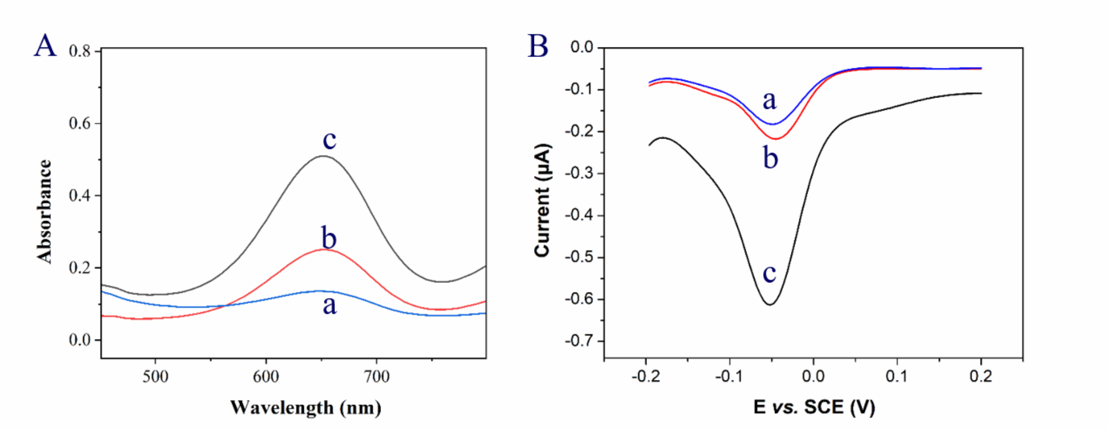

Figure S3. (A) Colorimetric sensor (B) Electrochemical sensor for detection of *P. aeruginosa* at different concentrations. *P. aeruginosa* analysis results from three different sensors: DNA+G4 (a), Single DNA+RCA (b), and DNA+RCA (c).

S4. The Linear Relationship of the Proposed Dual-mode Biosensor

As shown in Fig S4, the ideal calibration curves of electrochemical and colorimetric sensors were plotted. In the electrochemical channel (Fig S4A), the electrochemical biosensor for sensing *P. aeruginosa* has a good relationship in the range of 10-10^{7} CFU mL^{-1} with the limit of detection (LOD) of 1.7 CFU mL^{-1}.By contrast, the colorimetric sensor was a positive correlation (Fig. S4B) also showed a good linear relationship in the ranger of 1-10^{8} CFU mL^{-1} with a LOD of 1.3 CFU mL^{-1}. The equation applied to calculate LOD was $C_{(LOD)} = 10^{3\sigma b/slope}$, in which "σb" is the standard deviation of blank. The standard deviation of blank in electrochemical and colorimetric sensors were 0.0028 and 0.0015, individually.

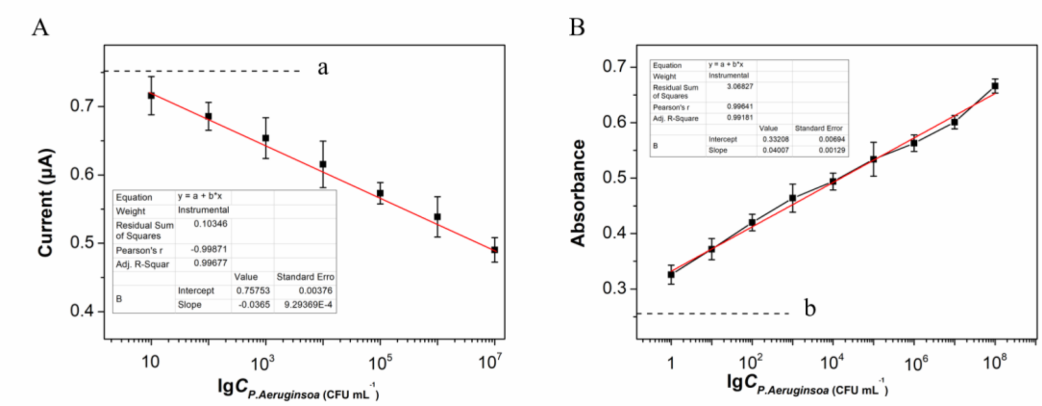

Figure S4. The insets in Fig 4A and B shows the linear relationship between the respective peak intensity and the patina concentration between 10-10^{7} CFU·mL^{-1} and 1-10^{8} CFU·mL^{-1}. The horizontal lines a, b represents the respective blank values. Error bars showed the standard deviation of three experiments.

S5. Stability of Colorimetric Sensor

Since the colorimetric sensor was detected by the naked eye, the choice of time was particularly important. For this purpose, the absorbance value changes over time were experimentally recorded. Fig S3 clarifies that after 8 min, there was no significant change.

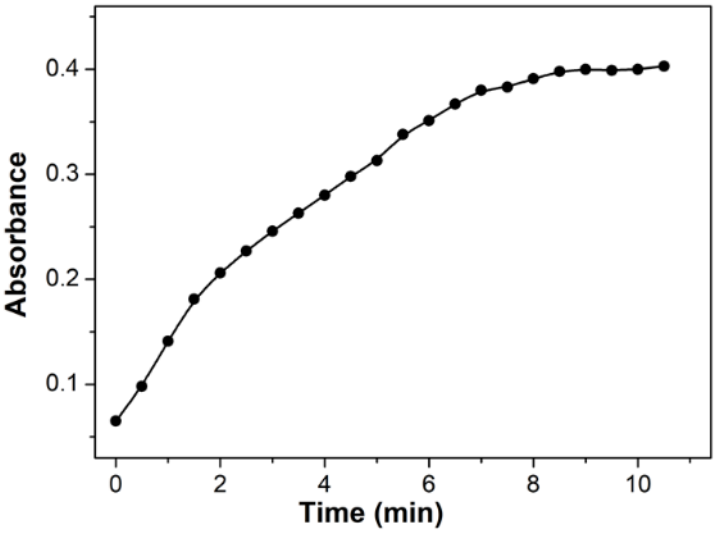

Figure S5. The relationship between absorbance and time in colorimetric sensor.

Reference

1. Yuan, W.; Lu, L.X.; Lu, Y.C.; Xiong, X.; Li, Y.; Cui, X.W.; Liu, Y.J.; Xiong, X.H. Synergistic effects of DNA structure for ultrasensitive detecting OTA in grains. Food Anal. Methods 2021, 14, 2308-2316. https://doi.org/10.1007/s12161-021-02060-x.
2. Xiong, X.H.; Yuan, W.; Li, Y.F.; Lu, Y.C.; Xiong, X.; Li, Y.; Liu, Y.J.; Lu, L.X. Sensitive electrochemical detection of aflatoxin B1 using DNA tetrahedron-nanostructure as substrate of antibody ordered assembly and template of aniline polymerization. Food Chem. 2020, 331, 127368. https://doi.org/10.1016/j.foodchem.2020.127368.
3. Lu, L.X.; Yuan, W.; Xiong, Q.; Wang, M.H.; Liu, Y.J.; Cao, M. Xiong, X.H. One-step grain pretreatment for ochratoxin A detection based on bipolar electrode-electrochemiluminescence biosensor. Anal. Chim. Acta 2021, 1141, 83-90. https://doi.org/10.1016/j.aca.2020.10.035.
4. Li, H.B.; Liu, M.B.; Zhao, W.H.; Pu, J.M.; Xu, J.G.; Wang, S.Q.; Yu, R.Q. Multi-channel collection of G-quadruplex transducers for amplified signaling of Pax-5 based on target-triggered split-to-intact remodeling of dual-G-rich duplex probe. Sens. Actuator B Chem. 2020, 311,127913. https://doi.org/10.1016/j.snb.2020.127913.
5. Yu, J.L.; Wu, H.H.; He, L.Y.; Tan, L.; Jia, Z.J.; Gan, N. The universal dual-mode aptasensor for simultaneous determination of different bacteria based on naked eyes and microfluidic-chip together with magnetic DNA encoded probes. Talanta, 2021, 225, 122062. https://doi.org/10.1016/j.talanta.2020.122062.
